# Age at initiation & prevalence of tobacco use among school children in Noida, India: A cross-sectional questionnaire based survey

**Published:** 2011-03

**Authors:** Raj Narain, Sarita Sardana, Sanjay Gupta, Ashok Sehgal

**Affiliations:** *Division of Epidemiology & Biostatistics & Preventive Oncology (ICMR), Noida, Uttar Pradesh, India*; * *Division of Cytopathology, Institute of Cytology & Preventive Oncology (ICMR), Noida, Uttar Pradesh, India*

**Keywords:** Age at initiation, prevalence, smoking, tobacco chewing, tobacco use

## Abstract

**Background & objectives:**

Tobacco use among school children is becoming a serious problem in developing countries. The early age of initiation underscores the urgent need to intervene and protect this vulnerable group from falling prey to this addiction. The present study was thus undertaken to assess the prevalence of tobacco habits among school children, determine the age of initiation of these habits, and compare the age of initiation between students who were more than 15 and ≤ 15 yr of age.

**Methods:**

Data on tobacco use were collected from 4786 students of class 7 to 12 (age: 11-19 yr) studying in different private and government schools of Noida city during July- December 2005, through cluster and random sampling using a self-administered questionnaire.

**Results:**

Any kind of tobacco use was found in 537 (11.2%) students; 419 (8.8%) were ‘ever smokers (including current smokers)’ 219 (4.6%) were ‘ever tobacco chewers (including current chewers)’, 179 (3.7%) were ‘exclusive smokers’ and 118 (2.5%) were ‘exclusive tobacco chewers’. The mean age of initiation of these habits was around 12.4 yr. More than 50 per cent of tobacco chewers reported use of khaini at least once. Nearly 70 per cent of boys and 80 per cent of girls ≤ 15 yr initiated the habit of tobacco before the age of 11 yr. A significant early uptake of tobacco chewing was reported from private school students as compared to government school students (*P*<0.05).

**Interpretation & conclusions:**

Tobacco addiction is emerging as a big threat among children. Our findings indicate a recent downward shift in the age at initiation of tobacco uptake and rising prevalence among girls. Such data need to be collected from different parts of the country to develop anti-tobacco campaigns and take policy decision.

Tobacco use is a leading cause of preventable deaths world over, more so in developing countries. In India alone, nearly 1 in 10 adolescents in the age group 13-15 yr have ever smoked cigarettes and almost half of these reports initiating tobacco use before 10 yr of age^1^.

The tobacco situation in India is unique because of a vast spectrum of tobacco products available for smoking as well as smokeless use^2^.Smoking of cigarette particularly beedis and chewing tobacco (smokeless use) is an age-old practice in India. However, according to anecdotal evidence with the changes in the dynamics of societies, the prevalence of smoking among women and young children has increased many folds and is at present a significant public health problem.

There are only a few studies on prevalence and initiation of smoking and smokeless tobacco use among children in our country^3^,^4^.The risks of tobacco use are highest among those who start early and continue its use for a long period^5^. The early age of initiation underscores the urgent need to intervene and protect this vulnerable group from falling prey to this addiction^6^. The most common reasons cited for children to start using tobacco are peer pressure, parental tobacco habits and pocket money given to children^7^. The present cross-sectional study was undertaken to determine the prevalence and age at initiation of tobacco smoking or tobacco chewing among school children in Noida city in north India. The specific objectives were (*i*) to assess the prevalence of tobacco habits among school children, (*ii*) to determine the age of initiation of these habits, and (*iii*) to compare the age of initiation between students who were more than 15 and ≤ 15 yr of age.

## Material & Methods

Information was collected from students of class 7 to 12 (age: 11-19 yr) studying in different schools of Noida city through a pre-tested, anonymous, closed and open-ended self-administered questionnaire during July- December 2005.

A cluster sample design was used to produce a representative sample of schools. At the first stage, a list of all Private (not funded by the government) and purely government aided schools having grades 7-12 was prepared. At the second stage, the schools were selected randomly from each area. The schools selected were representative of the types of schools in Noida city, *viz*., private, government girls only, government boys only and co-educational schools. The classes were randomly selected and from each selected class, all students of every alternate section were included. The study was approved by our ethics and review committee of Institute of Cytology & Preventive Oncology (ICMR), Noida. The principals of the schools were informed in writing about the importance of survey. Students were told to participate in the study voluntarily and an informed consent from the students and school authorities was obtained. Children were explained about how to fill up the questionnaire and to provide authentic information. They were assured that all information would be kept confidential. This health questionnaire about tobacco use was prepared based on questionnaire from Global Youth Tobacco Survey (GYTS)^8^. No changes were made in questions but some were excluded. The questionnaire was provided in English to Private school students and was translated in Hindi for Government school students. The translated version was validated before survey.

The data were collected on socio-demographic profile, occupation and literacy status of their parents. Data were also collected on use of tobacco, age at initiation, smoking habits of parents and siblings, peer influence, reason of initiation of tobacco, places of tobacco consumption, purchase of tobacco for elders at home and teachers, *etc*. ‘Ever use of tobacco’ was defined as the use of tobacco even once including current tobacco use^9^. Tobacco consumption was broadly classified into three categories: smoking, chewing and more than one form of tobacco use. Tobacco smoking includes cigarettes, beedis and others such as hookah, chillum, ganja, *etc*. Smokeless tobacco use includes Gutka, Khaini and Zarda^10^,^11^ (Appendix).

The target sample size calculated was 5600, keeping in mind the least prevalence of tobacco use as 6.0 per cent, a relative precision of 10 per cent and confidence interval of 95 per cent. A total of 5646 questionnaires were distributed to all eligible students in 17 schools of Noida. Those students who refused to participate were asked to remain in the class and not to disturb the participants.

Data were analyzed using Epi-info 6.04 D os0 version (free software downloaded from internet) and SPSS (version 11). Differences in proportions between the various groups *viz*., between girls and boys; between tobacco users and never users and differences in the type of tobacco used based on the type of schools (Private schools vs. Government schools) were tested using Pearson’s Chi-square test or Fisher’s exact test, as appropriate along with Odds ratio and 95 per cent confidence intervals (CIs). To compare the mean age of study variables, Students t-test was applied and alpha was set at 0.05.

## Results

A total of 4786 students (85.0% response rate) filled the questionnaire (boys: 49.3% and girls: 50.7%); of the remaining 860 students, some were absent on the day of collection while the remaining declined to participate because of parental refusal. The mean and median age for students was 14.8 ± 1.6 and 15 yr respectively. Almost an equal number of boys 2360 (49.3%) and girls 2426 (50.7%) responded and there was no difference in their mean ages (boys: 14.8 ± 1.6 and girls: 14.8 ± 1.5 yr). More than 90 per cent of the students belonged to Hindu families. The predominant occupation of the father was service, more than 55 per cent of them had educational status of high school and above and fathers of only 3 per cent students were illiterate. In contrast, majority of the mothers (73%) were housewives, about 50 per cent had studied up to or beyond high school and as high as 17 per cent were illiterate. About 70 per cent of responders resided in urban areas while 30 per cent resided in semi-urban settings. Government school students comprised 44 per cent (n=1914) of the subjects, while 2872 (56%) studied in private schools.

*Prevalence of ever use of tobacco:*‘Ever tobacco use’ in any form (smoking or chewing tobacco) was found in 537 (11.2%) students, of which 197 (4.1%) were current tobacco users. ‘Ever smokers’ comprised 419 (8.8%) [current smokers 148 (3.1%)], ‘ever tobacco chewers’ comprised 219 (4.6%) students (current tobacco chewers 1.9%). ‘Exclusive ever smokers’ comprised 6.6 per cent, ‘exclusive ever tobacco chewers’ 118 (2.5%) and ‘ever both smoking and tobacco chewing’ 101 (2.1%). Overall, the prevalence of tobacco use in any form was significantly more in boys as compared to girls (*P*<0.05). ‘Exclusive ever smoking’ was found to be equally common among boys and girls while ‘exclusive ever chewing’ and both habits (‘ever smoking and tobacco chewing’) were significantly more common among boys as compared to girls (Table I).

**Table I:** Prevalence of ever tobacco use among boys and girls

Habits	Sex		Total 4786	*P* value	O.R. (95 % C.I.#)
	Boys N=2360 user	Girls N=2426 user			

Exclusive smokers (A)	157 (6.7)	161 (6.6)	318 (6.6)	NS	1.0 (0.79-1.27)
Exclusive tobacco chewers (B)	73 (3.1)	45 (1.9)	118 (2.5)	<0.01	1.7 (1.12-2.47)
Use of both forms of tobacco (smoking and chewing simultaneously) (C)	60 (2.5)	41 (1.7)	101 (2.1)	<0.05	1.5 (1.0-2.31)
Ever smokers (A+C)	217 (9.2)	202 (8.3)	419 (8.8)	NS	1.1 (0.91-1.37)
Ever tobacco chewers (B+C)	133 (5.6)	86 (3.5)	219 (4.6)	<0.001	1.6 (1.22-2.15)
Ever tobacco users (smoking or chewing or both) (A+B+C)	290 (12.2)	247 (10.2)	537 (11.2)	<0.05	1.2 (1.02-1.48)
Never tobacco users	2070 (87.8)	2179 (89.8)	4249 (88.8)		

Figures in parentheses indicate prevalence per cent

# Prevalence in Girls taken as reference category

NS, not significant

Among all the students, 5.3 per cent (256 of 4786) had ever smoked cigarette exclusively (boys: 5.7%, girls: 5.0%). Girls had ever used zarda (10 of 2426; 0.4%) and smoked beedis (61 of 2426; 0.4%) more frequently than boys (52 of 2360; 2.2%) while among boys khaini chewing (44 of 2460; 1.9%) and habit of ‘chewing multiple products’ (46 of 2460; 1.9%) was more prevalent. When different smoking and chewing habits were analyzed independently, it was found that types of smoking and chewing tobacco did not differ according to gender (Table II).

**Table II:** Prevalence of ever use specific types of tobacco smoking and chewing by gender among ever users

I. Type of smoking (ever smokers, N=419)	Tobacco smoking boys n=217 (%)	Tobacco smoking girls n=202 (%)	Total tobacco smokers n=419 (%)

Beedi	52 (24.0)	61 (30.2)	113 (27.0)
Cigarette	134 (61.8)	122 (60.4)	256 (61.1)
Others (Hookah, Cigars, pipe, ganja and chillum,*etc.*)	13 (6.0)	9 (4.5)	22 (5.3)
Combined (Beedi & Cigarette)	18 (8.3)	10 (5.0)	28 (6.7)
Never tobacco smokers	2143 (90.8)	2224 (91.7)	4367 (91.2)
II. Type of tobacco chewing (ever tobacco chewers, N=219)	Tobacco chewing boys n= 133 (%)	Tobacco chewing girls n=86 (%)	Total tobacco chewers n=219 (%)
Gutka	37 (27.8)	23 (26.7)	60 (27.4)
Khaini	44 (33.1)	24 (27.9)	68 (31.1)
Zarda	6 (4.5)	10 (11.6)	16 (7.3)
Combined (Gutka & Khaini & zarda)	46 (34.6)	29 (33.7)	75 (34.3)
Never tobacco chewers	2227 (94.4)	2340 (96.5)	4567 (95.4)

‘Ever use of tobacco’ was found to be more in government school boys (13.4%) in comparison to private school boys (11.7%) whereas a reverse pattern was observed amongst girls (government:8.6%, private: 11.8%; *P*<0.01). ‘Exclusive smoking’ was found to be significantly more prevalent among both boys and girls of private schools in comparison to government school students (private: 121, government: 58 *P*<0.05).

*Age at first use (initiation)*: There was no significant gender difference in the age of uptake of overall tobacco habits (Table III). Nearly 70 per cent of boys and 80 per cent of girls up to the age of 15 yr initiated the habit of tobacco before the age of 11 yr. Among younger boys (≤15 yr), significantly more numbers initiated tobacco habits before 11 yr of age as compared to older boys (>15 yr) (*P*<0.01). Similar trend was observed in girls also (*P*<0.01). The uptake of tobacco habits ‘before age 11 yr’ was found to be 1.8 times earlier among girls than boys (*P*<0.01, CIs: 1.19-2.69). A statistically significant linear trend of age at initiation of any tobacco habit was observed in the age group of up to 15 yr, among both boys and girls (*P*<0.05 and *P*<0.01 respectively) (Table IV). Thirty five per cent of private school students initiated tobacco chewing before the age of 11 yr while only 14 per cent government school students initiated it at this age (*P*<0.05) (Table V).

**Table III:** Mean ages (yr) at initiation of different tobacco habits among ever using students

Habits	Boys n=2360	Girls n=2426	All students

Ever tobacco use	12.6 ± 2.0 (256)	12.3 ± 1.9 (221)	12.4 ± 1.9 (477)
Ever smokers	12.5 ± 1.95 (205)	12.3 ± 1.95 (191)	12.4 ± 1.95 (396)
Ever tobacco chewers	12.6 ± 2.0 (108)	12.1 ± 1.5 (68)	12.4 ± 1.8 (176)
Exclusive ever smoking	12.5 ± 1.9 (148)	12.4 ± 2.0 (153)	12.5 ± 2.0 (301)
Exclusive ever tobacco chewing	12.6 ± 2.0 (51)	12.0 ±1.0 (30)	12.4 ± 1.7 (81)
Use of both forms of tobacco (smoking and chewing simultaneously)	12.5 ± 2.1 (57)	12.2 ± 1.8 (38)	12.4 ± 1.9 (95)

No significant difference between boys and girls

**Table IV:** Distribution of ever tobacco using students with ‘any tobacco habit’ according to age of initiation and age group of the student

Age at initiation (in yr)	Boys				
	Age group			*P* value #	O.R. (95 % C.I.)
	≤15 yr (%)	>15 yr (%)	Total (%)		

<11	45 (69.2)	20 (30.8)	65 (25.2)	<0.01	2.2 (1.18 - 4.23)
12-13	63 (51.2)	60 (48.8)	123 (47.7)	NS	0.7 (0.44 - 1.25)
>14	34 (48.6)	36 (51.4)	70 (27.1)	NS	0.7 (0.39 - 1.26)
Total	142 (55.0)	116 (45.0)	258		
<11	66 (79.5)	17 (20.5)	Girls 83 (37.6)	<0.01	2.7 (1.35 - 5.25)
12-13	56 (62.9)	33 (37.1)	89 (40.3)	NS	0.7 (0.4 - 1.35)
>14	26 (53.1)	23 (46.9)	49 (22.2)	<0.05	0.5 (0.23 - 0.93)
Total	148 (67.0)	73 (33.0)	221		

# Reference category: Age >15 yr; O.R., Odds ratio (95% Confidence Interval)

NS, not significant

**Table V:** Age at initiation of different forms of tobacco use according to type of school

Age group at initiation (yr)	Exclusive ever smokers		Exclusive ever tobacco chewers		Both habits (smoking & tobacco chewing)	
	Types of schools		Types of schools		Types of schools	
	Private	Govt.	Private	Govt.	Private	Govt.

≤11	38 (35.9)	26 (45.6)	14* (35.0)	6 (14.0)	11 (23.4)	11 (22.9)
12-13	41 (38.7)	20 (35.1)	19 (47.5)	29 (67.4)	19 (40.4)	30 (62.5)
≥14	27 (25.5)	11 (19.3)	7 (17.5)	8 (18.6)	17 (36.2)	7 (14.6)
Total	106 (65.0)	57 (35.0)	40 (48.2)	43 (51.8)	47 (49.5)	48 50.5)
Mean ± SD	12.79 ± 1.56	12.47 ± 1.54	12.65 ± 1.42	13.1 ± 1.15	12.7 ± 2.4	12.1 ± 1.3
Median	13.0	13.0	13.0	13.0	12.0	12.0
Mode	13.0	11.0	13.0	13.0	12.0	12.0

* *P*<0.05 compared to Govt. school

Figures in parentheses indicate percentages

Govt. schools have been taken as baseline (reference group)

## Discussion

Majority of the tobacco related deaths occur in developing countries where problem of tobacco is assuming alarming proportions. A study at Gujarat State, India, showed a downward shift in age of initiation of this habit which is a matter of serious concern^12^.

The prevalence of tobacco use among school students in different States of India has been reported to vary from 1.9 per cent (Delhi) to 75.3 per cent (Mizoram)^3^,^4^,^13^–^18^.The prevalence of ‘ever use of tobacco’ in the present study was 11.2 per cent (boys: 12.2%, girls: 10.2%) which is much lower than the 37.2 per cent (boys: 39.2%, girls: 31.9%) reported by GYTS performed in Uttar Pradesh^5^. Lower prevalence inNoida city may actually reflect the rates of its neighbouring city of Delhi, as a large proportion of school students in Noida are residents of Delhi. Though tobacco use is on rise among girl students, we found the habit to be still a little less prevalent in girls than in boys. This observation is corroborated by other Indian studies^6^,^10^,^18^. The overall prevalence of ‘ever smoking’ (8.8%) in the present survey was also quite low in comparison to studies done in Haryana (12.0%)^18^ and Jamnagar (14.6%)^19^. According to GYTS 1999-2001, most countries *viz*. AFRO, AMRO/PAHO, EMRO, EURO, WPRO and SEARO also reported higher prevalence of ‘ever smoking’ varying from 10.2 to 73.6 per cent except Nepal where lower prevalence rate (6.5%) was reported^8^.

‘Ever use of tobacco’, particularly the use of smokeless tobacco products, was found to be more common in government school boys in comparison to private school boys, whereas this pattern was reverse amongst girls. This has considerable public health implication as it is known that people change over from smokeless form to smoking over a period of time. Our findings are in accordance with the results of Mathur *et al*^20^. Low socio-economic status of government school students may be responsible for this, since being relatively inexpensive and readily available, these children often see tobacco as an alternative to food^10^,^21^.Also chewing of these products is considered less harmful than smoking. Increasing practice of tobacco use in private schools may be attributed to peer pressure, rising influence of western culture, rapid urbanization, increased disposable income in the affluent families, tobacco use by their role models, parental habits, media promotion and best friends.

In the present study, though no significant differences were observed in the overall mean ages of initiation for ‘ever smokers’ and ‘ever tobacco chewers’, notable gender differences were found. The mean age at initiation of ‘exclusive smoking’ was significantly lower in girls as compared to boys. Ageat onset of smoking in our study was consistent with that observed in developed countries. The studies conducted among U.S. youths for ‘smoking by race and ethnicity’ and in Jerusalem school children also found the mean age at initiation to be around 12 yr^22^,^23^. A little higher age of initiation was found among Chinese Americans (boys: 12.8, girls: 13.1 yr), Non-Hispanic white minors (boys: 12.5, girls: 13.0 yr) and South African students (males: 15.16 and females: 14.98 yr)^23^–^25^.Among the Indian studies, the mean age of initiation of tobacco use has been found to vary from 8 to 15 yr^4^,^10^,^15^. The majority of the tobacco users worldwide have reportedly first tried tobacco prior to age 18^6^, some starting as young as 10 yr^26^.Our survey reflects a recent downward shift in age at uptake of tobacco habit among children. In a study from Kerala the mean age at initiation was 10.7 yr for boys ≤13 yr and 13.2 yr for boys ≥16 yr at the time of survey^27^. Even though tobacco use by small children and particularly girls is thought to be culturally unacceptable in Indian society^13^, in our study as many as 31 per cent of ever tobacco using students started using tobacco before the age of 11 yr. This figure is lower than that reported from the north-eastern States of India and GYTS report from Uttar Pradesh, where over 65 and 72 per cent of students respectively reported uptake at or before 10 yr of age^5^,^13^. Similar results have been reported from small studies done in rural areas of Gujarat, Tamil Nadu, Karnataka and Goa where one third to one half of children under the age of 10 yr experimented with smoking or smokeless tobacco in some form^16^,^28^. In a study conducted in Nairobi, 72 per cent students reported tobacco initiation between age 12 and 16 yr^29^. On the other hand, according to National Youth Tobacco Surveillance, United States, 4.9 to 14.3 per cent middle school and 4.9 to 14.3 per cent high school students of different States started cigarette smoking before 11 yr of age; for smokeless tobacco use, the figures varied from 1.2-10.2 per cent for middle schools and 1.3- 8.7 per cent for high schools^30^.

The response rate was 85 per cent in the present study. Since the characteristics of non-responders (15%) who were present in the class but did not answer the questionnaire were not studied, it would be an important limitation in interpretation of the findings of the study. Use of self-reported age of onset from a cross-sectional data is another limitation. Longitudinal studies have demonstrated that older adolescents tend to report a later age of smoking onset than younger ones^17^.The smoking behaviour mentioned in the questionnaire may differ from the actual smoking habit. The potential bias resulting from some students being absent on the day of survey is another limitation as these students might have higher rates of health risk behaviours.

In conclusion, the observations of the present survey indicate a downward shift in the age at uptake of tobacco habit by children and a rising prevalence among girls. More such surveys need to be carried out in other large cities of the country in order to build comprehensive database for future policy decisions on anti-tabocco campaigns.

## Appendix. Definitions of various tobacco habits and products^10^,^11^

**Table:**

Habits	Definitions (Notes)

Beedi	Beedi is a cheap smoking stick, handmade by rolling a dried, rectangular piece of temburni leaf*(Diospyros melanaxylon)*with 0.15-0.25 g of sun-dried, flaked tobacco filled into a conical shape and the roll is secured with a thread. The length of a beedi varies from 4.0-7.5 cm. Beedis are commercially available in small packets.
Cigarette	Cigarette smoking is the second most popular smoking form of tobacco used in India. The prevalence varies greatly among different geographic areas and subgroups such as rural-urban.
Hookah	Hookah (a hubble bubble Indian pipe) is an indigenous device, made out of wooden and metallic pipes, used for smoking tobacco. The tobacco smoke passes through water kept in a spherical receptacle, in which some aromatic substances may also be added. Hookah smoking is a common method of socializing among the village folk, especially in the northern and eastern parts of India.
Pipe	Pipe is a tube with a hollow bowl at one end used for smoking tobacco.
Chillum	Chillum is a conical clay-pipe of about 10 cm long. The narrow end is put inside the mouth, often wrapped in a wet cloth that acts as a filter. This is used to smoke tobacco alone or tobacco mixed with*ganja*(marijuana) in northern parts of the country.
Cigars	Cigars are made of air cured, fermented tobacco, usually in factories, and are generally expensive. Cigar smoking is predominately an urban practice.
Ganja	Marijuana, the most commonly used illicit drug; considered a soft drug prepared from the flowering tops and leaves of the hemp plant; smoked or chewed for euphoric effect.
Charas	Charas is the name given to hand-made hashish in Afghanistan, Pakistan, Nepal and India. It is made from the extract of the cannabis plant*(Cannabis sativa).*
Gutka	Gutka is a manufactured smokeless tobacco product (MSTP), a mixture of areca nut, tobacco and some condiments, marketed in different flavours in colourful pouches.
Khaini	Khaini consists of roasted tobacco flakes mixed with slaked lime. This mixture is prepared by the user keeping the ingredients on the left palm and rubbing it with the right. The prepared pinch is kept in the lower labial or buccal sulcus. Its use is common in eastern India.
Zarda	Zarda is hygienically processed & packed chewing tobacco.
